# The effect of scenario-based training on the Core competencies of nursing students: a semi-experimental study

**DOI:** 10.1186/s12912-023-01442-2

**Published:** 2023-12-13

**Authors:** Mohammad Sadeghi, Monirsadat Nematollahi, Jamileh Farokhzadian, Zohreh Khoshnood, Mostafa Eghbalian

**Affiliations:** 1 Razi Faculty of Nursing and Midwifery, Kerman University of Medical Sciences, Kerman, Iran; 2 Nursing research center, Kerman University of Medical Sciences, Medical University Campus, Haft-Bagh Highway, Kerman, 7616913555 Iran; 3 Department of Community Health Nursing, Razi Faculty of Nursing and Midwifery, Kerman University of Medical Sciences, Kerman, Iran; 4 Endocrinology and metabolism research center, Institute of basic and clinical physiology sciences, Kerman University of Medical Science, Kerman, Iran

**Keywords:** Core competencies, Scenario, Scenario-based education, Nursing students

## Abstract

**Introduction:**

Competency is defined as the variety of skills and knowledge required to perform a specific task. Due to the specificity of pediatric nursing, students face some challenges in acquiring core competencies. Therefore, the use of new training methods in pediatric nursing is necessary. One of the modern learning methods is learning based on clinical scenarios. Thus, this study aimed to investigate the effect of scenario-based education on the core competencies of nursing students.

**Method:**

This quasi-experimental study employed a pre-test and post-test design. All participants (n = 72) were selected via the census method and randomly divided into intervention (N = 33) and control groups (N = 40). The data were collected using a demographic information questionnaire and the Nursing Students’ Clinical Competencies Questionnaire. Before the intervention, both groups completed the pre-tests. After one month, the students in both groups completed post-tests.

**Results:**

The average score of core competencies for the students in the intervention group after the training (247.05, SD = 36.48) increased compared to before the intervention (229.05, SD = 36.58) (P > 0.05). The average score of the core competencies for the students in the control group after the training was 240.76 (SD = 35.36) compared to 235.56 (SD = 27.94) before the intervention, with no significant difference (P < 0.05). The independent t-test did not show a significant difference between the control and intervention groups before and after the intervention (P > 0.05).

**Conclusion:**

The results indicated the effectiveness of scenario-based training on the core competencies of students in the intervention group. Accordingly, nursing administrators and professors are recommended to incorporate new scenario-based teaching and learning methods in educational programs of universities. It is also necessary to conduct more research into the effectiveness of this method in combination with other training methods like team-based and problem-based training.

## Introduction

Competence reflects an individual’s actions and abilities to fulfil job responsibilities. In contrast, competency represents an individual’s performance in specific circumstances to implement successfully. Competency has always been the core goal of nursing, in contrast to other aspects of healthcare in which getting the job done has always been the primary focus [1]. In nursing, core competencies are defined as a set of skills or procedures that an individual needs to perform a practice successfully or competently [2]. These competencies include eight components of critical thinking, general clinical skills, basic medical sciences, communication and teamwork, care, ethics, responsibility, and continuous learning [3].

The efficiency of the nursing system of any country depends on nurses’ competencies. That’s why this concept has received much attention in the last century. Thus, many developed countries use different approaches and techniques such as continuing education, theoretical and practical tests, and working hours. Moreover, obtaining scientific degrees improve nurses’ clinical competence [4]. The competence of novice nurses as the output of nursing education in professional courses is essential in providing safe care for patients in the complex hospital environments [5].

Studies indicate that the effectiveness of undergraduate nursing education is not enough, and novice nurses have not been able to get the necessary preparation for the factual environment of care [6, 7]. Willman et al. showed that critical thinking competency one of the core competencies received the lowest self-assessed score among the newly graduated nurses [8]. Studies in Iran also show that novice nurses who start their professional role do not have sufficient cognitive skills to fit the needs of factual clinical environments [9, 10]. Abedi et al. also listed cases such as unpreparedness in the clinical role, weak professional competence, lack of self-confidence, inability to meet colleagues’ expectations, and adverse emotional reactions as some challenges faced by a majority of nursing students [11]. Challenges identified in various studies on newly graduated nurses entering the workplace include transition problems (from education to work), overload, poor organization of patient care, and inadequate responses to patient problems due to a lack of competency [12].

Children’s physical functions are underdeveloped compared to adults, putting children at high risk of negative consequences from infections and injuries. Therefore, proper and very accurate nursing care should be provided to young patients [13]. Nursing students must acquire sufficient competence through education to identify and solve nursing problems related to pediatric patients and their families and promote the health of children and their families in clinical environments after graduation [14].

Many clinical educators are looking for educational methods to teach clinical knowledge and skills effectively to nursing students. The best way to achieve this goal is to use a training method in which students are active in learning and can receive proper feedback about their learning [15].

Many researchers have stated that scenario-based teaching is a much more dynamic interactive teaching and learning strategy [16, 17]. In addition, many authors emphasized that this method mainly focuses on enhancing students’ abilities and skills, including analytical, thinking, problem-solving, communication, and teamwork skills required in the 21st century [18, 19]. Also, it improves students’ ability and self-confidence [20, 21]. The scenario-based learning method can help students think critically and enhance their decision-making skills and self-directed learning abilities [22].

The result of a study by Yang (2018) showed that the clinical education of pediatric nursing using the scenario simulation method significantly increased the theoretical and practical scores of students in the evaluation by the instructor compared to the control group [23]. Uysal (2016) also showed that implementing scenario-based education could reduce the common mistakes made by students in the practical exam each year compared to the previous year and students also stated that scenario-based learning facilitates learning and knowledge retrieval [24]. Izadi et al. (2020) also reported that nursing ethics training using scenario-based and discussion groups could significantly improve the observance of patient rights and patient satisfaction with nurses immediately and one month after the intervention [25]. Du et al. (2021) reported that clinical scenario-based simulation training improved students’ competencies in bed sore identification, disease prevention during hospitalization, and disease worsening prevention (rehabilitation) [26].

Pediatric simulations allow students to apply knowledge gained in an educational setting to a clinical scenario and receive teachers’ feedback [27, 28]. Lubbers and Rossman reported that undergraduate nursing students who participated in pediatric simulations reported higher levels of self-confidence and satisfaction after their simulation experiences [29, 30]. Similarly, Parker and colleagues found that undergraduate students in a pediatric simulation gained more confidence in clinical skills and were pleased with their participation in medium- and high-fidelity clinical simulations [28]. Karageorge et al. (2020) showed that implementing scenario simulation could improve the knowledge, skills, and self-confidence of pediatric intensive care nurses in the face of a crisis [31].

A review of the literature showed that scenario-based training could have profound effects on improving the various dimensions of nursing competencies. It also improves the student’s efficiency and clinical performance in pediatric care. Since the studies conducted in Iran indicated the undesirable quality of services, also, care provision for patients was not based on scientific evidences and no study has addressed the impact of the scenario-based method on the core competencies of students, the present study aims to investigate the effect of scenario-based education on the core competencies of nursing students of the Nursing Faculty of Kerman University of Medical Sciences in Iran.

## Methodology

The aim of this study was to investigate the effect of scenario-based training on the core competencies of nursing students.

**Fig. 1:**
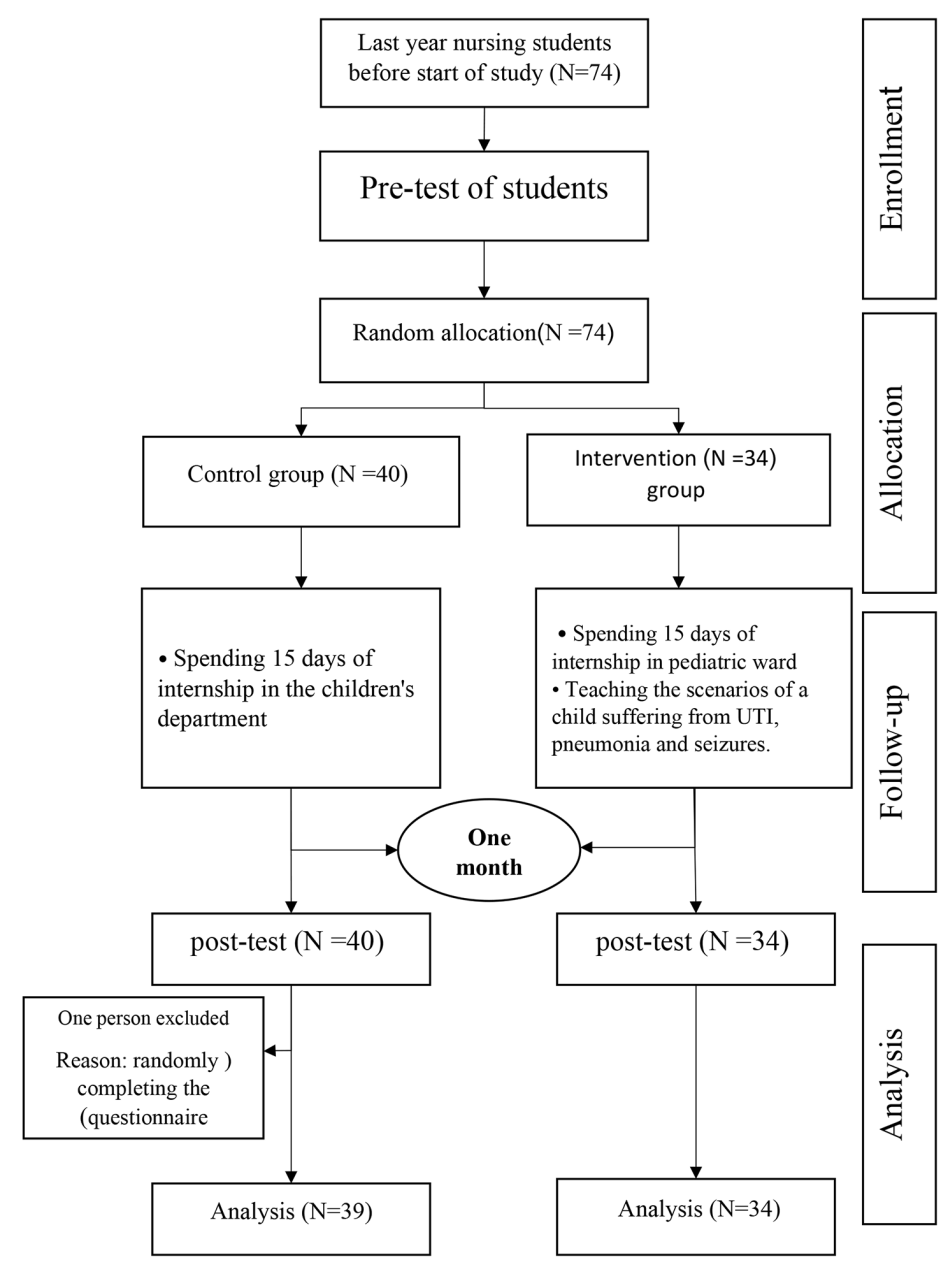
Flow chart of the study

This quasi-experimental study was conducted using a pre-test and post-test design in the Faculty of Nursing and Midwifery affiliated with Kerman University of Medical Sciences in 2021, the protocol for the study was approved by the ethics committee of Kerman University of Medical Science (IR. KMU. REC. 1400. 385). All nursing interns who had passed the pre-internship theory exam and OSCE^1^ were selected by the census method and participated in the present study (N = 74).First the list of students and their student numbers was taken from the education department of the faculty and entered the study in the same year. All of the students were listed randomly by lottery method, then the internship complete with the sequence of students in the list so that the first students of the list completed the first semester internship (N = 40) and other students (N = 34) enrolled the study in second semester. One student was excluded from the study, and finally, the data of 73 students (control = 39 and intervention = 34) were analyzed. To prevent information exchange between the two groups, at first, the control group had included in the study, and the training of the intervention group began when the control group had completed their post-tests (Fig. 1).

Afterward, two groups were formed in the WhatsApp application. the study objectives were explained to the students in both groups. Their informed consent to participate in the study had obtained, all students received information regarding the study and signed consent. Participation was voluntary, and students had the right to withdraw without adverse effects on their academic standing. The confidentiality and anonymity of personal data were maintained by coding students’ identity. Then nursing students’ core competency questionnaire (NSCCQ) was completed by the participants in the two groups.

After completing the NSCCQ, the students in the control group attended the pediatric ward in group of 8 based the arrange of students in the randomized list for 15 days according to the usual clinical training protocol in the faculty during the first three days. The rules and regulations were explained to the students and the conventional care protocol was implemented by the supervisor. Then, from the fourth day, the patient was handed over to students. At this time, the students of the control group observed the cases of seizure, urinary infection, and pneumonia, and they provided care to the patient under the supervision of the supervisor. This process continued until the fifteenth day under the supervision of the head of the pediatric ward. The students in the control group provided routine clinical care for the patients using a case method form, and after one month, they were asked to complete the NSCCQ again.

The students in the intervention group attended the pediatric ward in groups of 8 people based on list order. On the first day of the internship, the head nurse of the ward, who was also responsible for students’ clinical training, introduced the prevalent cases in the department to the students. They were required to obtain information about the presented cases (1- a child with seizures, 2- a child with a urinary infection, 3- a child with pneumonia). The students were required to check the clinical symptoms of the affected child, taking history, following up the process of diagnosis and treatment of the disease, and providing nursing care to the patients in the first ten days of the internship. During these ten days, a senior pediatric nursing expert was present in the department as a mentor for one hour and answered the student’s questions regarding the documentation of the case and other questions. After ten days of internship in the pediatric ward, the students participated in the Skyroom course in a group of eight that they were. Each scenario-based training session was held online for 2 h for the students the intervention group. The scenarios of seizure, urinary infection, and pneumonia were presented to the intervention group through video, pictures, and group discussion. The course instructors were a pediatric nursing doctor and a nursing master’s student. In designing the scenarios, the simulated cases were discussed from simple to complex.

At the beginning of the online classes, the information about the objectives, lesson plan, the duties of the students, and the title of the course was provided to the students. The scenarios were pre-written and approved by the professors of the pediatric department for content validity. the quality of scenario-based training controlled by two experts in pediatric diseases. Each case was discussed in a session. The scenarios were written in advance and were approved for content validity by the professors of the pediatrics department. In each scenario, a complete history of the patient was read by the senior student, and then questions were asked step by step about the proposed scenarios. Each student reported the actions they should take in dealing with this scenario, and then the professor examined and analyzed the answers to the students’ questions one by one. Afterward, the videos prepared for each scenario were shown, and the necessary actions when facing each scenario were also explained. Then, one of the latest articles that presented new findings related to the problem in the scenario was reviewed. In the end, the teacher summarized the whole discussion presented in the class. The reason for conducting the scenario discussion sessions in the last four days of the internship was to familiarize the students with the cases of the ward and help them better analyze the simulated scenarios. This helped students to imagine the designed scenarios and analyze the designed situation effectively. After one month, the students were asked to complete the NSCCQ again.

The data in this study were collected using a demographic information questionnaire and the Nursing Students’ Core Competencies Questionnaire.

The demographic information questionnaire assessed the students’ age, sex, marital status, native status, year of entering university, residence status, whether they have completed a practical nursing course, their interest in the nursing profession, number of past units, grade point average (GPA), and whether they were familiar with the scenario-based training method or not?

The competencies of all students in two groups were evaluated after one month as a post-test using a Questionnaire evaluating core competencies of nursing students developed by Perng and Watson. The questionnaire had 48 items, which measured eight dimensions of competencies: critical thinking, general clinical skills, basic medical sciences, communication skills, care, ethics, responsibility, and continuous learning. Each domain was measured using six items on a 7-item Likert scale from completely disabled to completely capable (1 = completely not capable; 2 = disabled; 3 = relatively disabled; 4 = medium; 5 = relatively capable; 6 = capable; 7 = completely capable). Total scores for 48 items ranged from 48 to 336, with higher the score students indicating higher competencies [3].

The questionnaire was translated and retranslated into Farsi by the researcher, and its content validity was measured quantitatively and qualitatively by ten professors of the Kerman University of Medical Sciences. For this purpose, a 4-point Likert scale was used (1 = not relevant, delete the item; 2 = somewhat relevant, need much revision; 3 = quite relevant, need a little revision; and 4 = very relevant). Its content validity coefficient was obtained as CVI^2^ = 1, and its reliability was measured by calculating the internal correlation coefficient and Cronbach’s alpha in a group of 40 students, and Cronbach’s alpha coefficient was 0.94. The results confirmed the reliability and validity of the instrument. The collected data were analyzed with SPSS26 software using the chi-square test, paired samples t-test, and independent samples t-test. The Kolmogorov-Smirnov test was used to check the normality of the data, and the significant level was considered p < 0.05.

### Ethical approval

was granted by the Research Ethic Committee of University of Kerman (IR. KMU. REC. 1400. 385). Research team confirm that all methods were carried out in accordance with relevant guidelines and regulations. Also, informed consent was obtained from all subjects.

## Results

In this study, both intervention and control groups were homogeneous in terms of all demographic variables, including age, sex, marital status, students’ grade point average, etc., and there was no significant difference between the two groups (p < 0.05) (Table 1). A large number of participants in the study (38 persons; 52%) were women. The average age of the students in the intervention group was 22.5 years (SD = 1.07), and the average age of the students in the control group was 22.3 years (SD = 1.61). The average GPA in the intervention group was 16.20 (SD = 1.40), and that of the control group was 16.38 (SD = 0.84).

**Table 1:** Comparison of qualitative demographic characteristics in control and intervention groups

variable	grouping	Intervention group	Control group	statistic	p-value (a)
		Frequency (%)	Frequency (%)		
gender	Male	50.0))17	18 (46.2)	0.10	0.816
	Female	17 (50.0)	21 (53.8)		
Marital status	Single	82.4)) 28	31 (79.5)	0.89	0.640
	married Others	6 (17.6) 0 (0.0)	7 (17.9) 1 (1.4)		
Living in Kerman?	Yes	19 (55.9)	22 (56.4)	0.002	0.575
	No	15 (44.1)	17 (43.6)		
year of entering University	2015 2016 2017	1 (2.9) 5 (14.7) 28 (82.4)	0 (0.0) 3 (7.7) 36 (92.3)	2.16	0.338
residence status	Dormitory With family alone	17 (50.0) 16 (47.1) 1 (2.9)	23 (58.9) 15 (38.5) 1 (2.6)	0.59	0.744
completed Practical nursing course	Yes No	2 (5.9) 32 (94.1)	3 (7.7) 38 (92.3)	0.09	0.566
level of interest in nursing	Very little Little Moderate Much Very much	3 (8.8) 4 (11.8) 9 (26.5) 9 (26.5) 9 (26.5)	0.0 (0.0) 4 (10.3) 17 (43.6) 11 (28.2) 7 (17.9)	5.59	0.231
Knowing scenario method	No Somewhat Yes	21 (61.8) 7 (20.6) 6 (17.6)	24 (61.5) 13 (33.3) 2 (5.1)	3.67	0.159

(a) chi-square/fisher exact test

According to the results, the average score of core competencies of the students in the intervention group increased from 229.05 (SD = 36.58) in the pre-test to 247.05 (SD = 36.48) in the post-test, showing a significant difference (P > 0.001). In the intervention group, a significant improvement was observed in all dimensions of competence except critical thinking and ethics in the post-test (P < 0.05). In the control group, there was no significant difference between the students’ core competency scores in the pre-test 235.56 (SD = 27.94) and the post-test 240.76 (SD = 35.36) (p < 0.05). Moreover, no significant improvement was found in any of the competency dimensions in the control group, except for the basic science dimension (p < 0.05) (Table 2).

**Table 2:** Comparison of the score of core competencies in the intervention and control groups, before and after the intervention

Type of competency	Groups	Mean ± SD Before	Mean ± SD After	Effect size	Statistic (P-value) (b)
	Independent test results				
Critical thinking	Control Intervention	4.39 ± 28.48 5.47 ± 28.00	5.04 ± 28.71 5.41 ± 29.26	0.04 0.27	-0.29 (0.772) 1.59 (0.121)
	**Statistic** **(P-value) (c)**	− 0.42 0.675	0.44 0.657		
Clinical skills	Control Intervention	4.42 ± 27.74 5.84 ± 26.97	28.33 ± 5.12 5.58 ± 29.29	0.10 0.59	-0.677 (0.509) -3.49 (0.001)
	**Statistic** **(P-value) (c)**	− 0.64 0.523	0.76 0.446		
basic medical sciences	Control Intervention	3.14 ± 26.56 5.58 ± 26.32	5.21 ± 28.64 5.25 ± 28.82	0.42 0.72	-2.64 (0.012) -4.21 (0.001)
	**Statistic** **(P-value) (c)**	− 0.22 0.825	0.14 0.882		
Communication skills	Control Intervention	4.30 ± 28.58 5.52 ± 28.11	5.50 ± 28.84 4.73 ± 31.26	0.04 0.77	-0.30 (0.762) -4.50 (0.001)
	**Statistic** **(P-value) (c)**	− 0.41 0.683	1.99 0.050		
Care	Control Intervention	5.05 ± 29.30 5.13 ± 28.44	5.14 ± 30.48 4.88 ± 31.82	0.25 0.77	-1.58 (0.122) -4.51 (0.001)
	**Statistic** **(P-value) (c)**	− 0.72 0.471	1.13 0.261		
Ethics	Control Intervention	5.42 ± 32.15 5.58 ± 31.38	5.74 ± 32.28 5.36 ± 32.70	0.02 0.27	-0.13 (0.892) -1.61 (0.116)
	**Statistic** **(P-value) (c)**	− 0.59 0.552	0.32 0.747		
Responsibility	Control Intervention	4.80 ± 32.00 5.02 ± 30.75	4.99 ± 32.56 5.18 ± 32.50	0.10 0.50	-0.65 (0.519) -2.92 (0.006)
	**Statistic** **(P-value) (c)**	− 1.09 0.276	-0.54 0.957		
Continuous learning	Control Intervention	4.48 ± 30.71 4.84 ± 29.08	4.93 ± 30.89 5.26 ± 31.38	0.03 0.60	-0.20 (0.839) -3.51 (0.001)
	**Statistic** **(P-value) (c)**	− 1.49 0.140	0.40 0.686		
Core competencies	Control Intervention	27.94 ± 235.56 36.58 ± 229.05	35.36 ± 240.76 36.48 ± 247.05	0.16 0.72	-1.03 (0.308) -4.21(0.001)
	**Statistic** **(P-value) (c)**	− 0.86 0.393	0.74 0.458		

(b) **Paired t-test**

(c) Independent t-test

The independent t-test between the control and intervention groups in the pre-test and post-test stages did not show any significant difference in any of the dimensions of the core competencies and in its overall score (pretest: TS^3^ = 0.86, P < 0.05, posttest: TS = 0.74, P < 0.05) (Table 2).

## Discussion

The study results showed that scenario-based training significantly improved the core competencies of the nursing students in the intervention group. Furthermore, this increase was significant in all dimensions of clinical skills, basic sciences, communication and teamwork, care, responsibility, and continuous learning, except for the two dimensions of ethics and critical thinking. Currently, not much research had found on the effectiveness of scenario-based education on the core competencies of nursing students.

Karageorge et al. (2020) assessed the skills of crisis management pediatric ICU nurses. The results showed that the simulated scenarios could improve the skills of pediatric ICU nurses including teamwork, knowledge, and self-confidence in the face of severe child health conditions [33]. The results of this study were consistent with the communication skills and basic medical science dimension of the current study that showed scenario-based training could improve the core competencies of nurses. However, the training intervention was conducted on a computer dummy.

Similarly, Pinar et al. (2016) investigated the impact of scenario-based simulation training on nursing students’ knowledge and childbirth skills. In this study, 23 nursing students underwent scenario-based simulation training related to maternal nursing and PowerPoint presentation. The results showed that the score of nursing knowledge and skills of the students in the intervention group increased [32]. The results of this study were consistent with the teamwork and clinical skills dimension of the current study. In this study, the intended scenarios were presented to the students as simulated cases on an obstetrics emergency simulator. The students had to practically perform the necessary actions learned previously during the lecture and PowerPoint. One of the limitations of this study the failure to measure all competencies and the small number of the participants, which was compensated in the present study.

Baek et al. (2022) reported that scenario-based learning improved team efficiency, systems thinking, and proactivity in problem-solving in nursing students without experience in nursing practicum [35]. The results of this study were consistent with the data on the critical thinking and communication skills dimension in the present study. The difference between this study and the current study was that the students themselves discovered possible scenarios that might arise in providing care, reached a consensus about them, and finally implemented them. Moreover, the participants in this study were last-year students.

Izadi et al. (2020) showed that scenario-based learning and group discussion made nurses comply with ethical codes and improved patients’ satisfaction with nurses’ performance [27]. In contrast to the present study, the educational intervention could significantly increase ethical performance as the scenario-based method was used in combination with group discussion, and all the training time had was dedicated to ethical issues.

Our results showed that the overall score of the core competencies in the control group did not increase significantly in the post-test. A significant improvement was observed only in the dimension of basic sciences in the control group. Similarly, Izadi et al. (2020) found no significant difference in the control group in adherence to ethical codes and patients’ satisfaction with nurses’ performance in the pre-test and post-test stages [25].

Delnavaz et al. (2018) showed that after applying the training through lectures and PowerPoint presentations, students’ knowledge and performance in the control group increased significantly [33]. This finding was inconsistent with the data in clinical skill dimension of the present study because the instructors taught triage using scenarios in a lecture method, checked the same items in the post-test, and did not measure all dimensions of competence.

The results showed no significant difference between the two groups in terms of core competencies and their dimensions.

Contrary to the findings of the present study, Yang (2018) found that clinical training in pediatric nursing students using the scenario simulation method could increase the clinical and theoretical performance scores of nursing students compared to the control group [23]. These conflicting results were probably because the students in the intervention group practically performed the intended roles and continued until mastery.

Jeong et al. (2022) showed that virtual reality simulation training based on the COVID-19 scenario was efficient. In both the control and intervention groups, significant differences were found in the nursing students’ knowledge about infectious respiratory diseases, self-efficacy, and clinical reasoning between the pre-test and post-test. However, no difference was found between the two groups [34], as was the case in the present study.

Du et al. (2021) showed that the OSCE scores of the nursing students in intervention group in the clinical scenario simulation to assess the risk of pressure ulcers were much higher than the control group [26]. The results of this study were not consistent with the present study because, probably, in the present study, the training sessions were held online, but in this study, face-to-face training was provided using simulated patients.

Liu and Xiao (2021) investigated the value of using the scenario simulation training method along with the progressive teaching mode and showed that the performance evaluation scores, basic knowledge, nurse-patient interaction, critical thinking, and comprehensive evaluation in the intervention group were significantly better than the control group [35]. The result of this study was inconsistent with the dimension of basic medical science, communication skills, and critical thinking of the present study in terms of the significance of the post-tests. The greater effectiveness of the intervention in this study was probably because of the integrated teaching method used in this study. First, theoretical and practical materials were taught individually, from simple to advanced, and then the students played the roles of patients, nurses, family members, etc., in the form of intended scenarios.

Zhang et al. (2021) showed that clinical skills in the intervention group improved significantly compared to the control group after using hierarchical training with simulation scenario training for operating room nurses [36]. This study was inconsistent with the clinical skills dimension of the present study, probably because the scenarios were performed face-to-face and in the hospital.

## Conclusion

This research results showed that scenario-based training in the virtual platform could significantly increase the core competencies of nursing students. The findings indicated that scenario-based training improves clinical skill competencies, basic science knowledge, communication and teamwork, care, responsibility, and continuous learning. In this way, students can promote the quality of services they provide to their community and provide safe care for children. This teaching method brings students closer to reality. It also engages them with what they encounter in practice. A result, learning moves from passive and traditional learning to active learning, which improves their core competencies and reduces the theory-practice gap. Thus, nursing managers and educational planners can use this educational method for future nurses. One of the limitations of the present study was the used of self-report instruments. Hence, the students tended to overestimate their competences. Therefore, students were asked to complete the questionnaires with sufficient accuracy.

One of the strengths of this study was measuring core competencies that were not much considered in research, although corona pandemic and online classes were a limitation. Better Consolidation of information in memory and deeper learning is another implication. Also, increasing nurses’ efficiency and performance causes More quality services. The results of this study can be used as a guide in future research design.

## Data Availability

All data generated or analyzed during this study are included in this published article.

## Footnotes

1 Objective structured clinical examination.

2 Content validity index.

3 T statistic.
